# Combining expression of RNF43 and infiltration level of CD163 ^+^ tumor associated macrophage predicts prognosis of clear cell renal cell carcinoma

**DOI:** 10.1002/cam4.5229

**Published:** 2022-09-12

**Authors:** Dawei Zhu, Xiaokai Shi, Yijun Tian, Hao Li, Bowen Tang, Ziyi Zhang, Ze Zhang, Li Zuo

**Affiliations:** ^1^ Department of Urology The Affiliated Changzhou No. 2 People's Hospital of Nanjing Medical University Changzhou China; ^2^ Department of Urinary Surgery The Third Affiliated Hospital of Naval Medical University Shanghai China; ^3^ Department of Graduate School Dalian Medical University Dalian China

**Keywords:** cancer risk factors, macrophages, microenvironment, renal cancer

## Abstract

Searching for reliable indicators for evaluating prognosis diagnosed with clear cell renal cell carcinoma (ccRCC) is crucial for improving clinical therapies. However, current researches have looked mainly at the prognostic value of a single intratumoral indicator, neglecting tumor‐infiltrating immune cells (TIICs) in the microenvironment. This study examined whether the integration of Ring finger protein 43 (RNF43) expression and CD163^+^ tumor‐associated macrophage (TAM) infiltration in combination with clinical indexes forecast ccRCC patient outcome with relatively high accuracy. Firstly, the expression of RNF43 and CD163 were detected with immunohistochemistry. Totally, 346 ccRCC patients were random separated evenly into training and validation datasets to make further analyses. We found that RNF43 expression was negatively correlated with infiltration level of CD163^+^ TAM in ccRCC, which was closely associated with the TNM stage and outcome of these patients. The multiple regression analysis demonstrated that RNF43, CD163, and TNM stage could function as independent risk factors in overall survival (OS) and progression‐free survival (PFS) prediction of ccRCC. Furthermore, a better postoperative prognosis index for ccRCC patients was obtained by combining RNF43 and CD163^+^ TAMs, which assessed with time‐dependent C‐index analyses and a nomogram. Consequently, combining RNF43 and CD163^+^ TAMs along with TNM stage acquired robust accuracy in forecasting outcome of patients with ccRCC. In conclusion, combining intratumoral RNF43 expression, CD163^+^ TAM infiltration, and TNM stage could significantly enhance the veracity in forecasting postoperative outcomes.

## Graphical abstract

Searching for reliable indicators for evaluating prognosis diagnosed with clear cell renal cell carcinoma (ccRCC) is crucial for improving clinical therapies. Our study has demonstrated that combining intratumoral RNF43 expression, CD163^+^ TAM infiltration, and TNM stage could significantly enhance the veracity in forecasting postoperative outcomes.

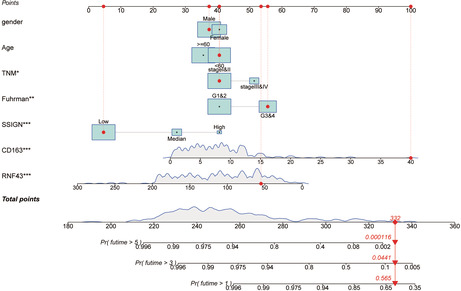

## 1 INTRODUCTION

In terms of renal cell carcinoma, the most prevalent kind is clear cell renal cell carcinoma, which is one of the world's most common forms of cancer.^1^, ^2^ Although operation is the main preferred treatment, the postoperative progression and recurrence of ccRCC usually occurs.^3^ In the clinic, TNM stage serves as the crucial indicator for evaluating and follow‐up of ccRCC patients.^4^ But some of ccRCC patients with low TNM stage still harbor a poor prognosis, and ccRCC patients with high TNM stage partially possess a long survival, which suggests the blind area of TNM stage.^5^ Thus, it is necessary to identify novel and authentic prognostic indicators in accurately predicting ccRCC patients' prognosis by combining with the established clinical indicators like TNM stage.

Many studies have shown that intratumoral biomarkers like oncogenes, tumor suppressors, lncRNA, etc. can be used to predict ccRCC patients' prognosis.^6^, ^7^, ^8^ But most of these studies only focused on the prognostic value of a single indicator, and did not combine current clinicopathological indicators such as tumor stage. A growing number of studies also show that tumor growth and development is dependent on communication between cancer cells and tumor‐associated macrophages (TAMs) in the microenvironment, in addition to the intratumoral signaling pathway.^9^, ^10^ Therefore, combining intratumoral markers, TAMs, and TNM stage might more comprehensively evaluate postoperative outcome of ccRCC patients.

Ring finger protein 43 (RNF43), a tumor suppressor and possible prognostic biomarker in ccRCC, has been identified in our recent studies.^11^ RNF43 expression and CD163 expression were shown to be negatively associated in ccRCC specimens, according to a preliminary bioinformatics analysis of Gene Expression Profiling Interactive Analysis (GEPIA)‐derived data. However, whether combining intratumoral RNF43 expression with TAMs could increase the prognostic accuracy for ccRCC patients by combining with the current clinical indicators like TNM stage remain unclear. Therefore, in the present study, 346 ccRCC patients were employed and separated evenly into training cohort and validation cohort. Subsequently, the predictive accuracy of combining RNF43, CD163^+^ TAMs, and TNM stage were investigated with statistical analyses in ccRCC.

## 2 MATERIALS AND METHODS

### 2.1 Patients

Totally, 346 individuals pathologically diagnosed with ccRCC between 2010 and 2014 were included in this research (Table S1). The research was conducted in accordance with the guidelines for tumor biomarker prognostic studies (REMARK).^12^ In addition, the R platform's sample function was utilized to randomly divide all patients into a training cohort (*n* = 173) and a validation cohort (*n* = 173). The size parameter was adjusted to 0.5 to ensure half of all samples into the training cohort, and the replace parameter in this function was set to FALSE to guarantee no samples were replaced during sampling. The average age at the time of diagnosis of patients screened in the cohort was used as a cut‐off value of age, which was (60.41 ± 10.76). Two of the most primary outcomes were overall survival (OS) and progression free survival (PFS). OS was defined as the time period from the time of surgery to the time of death or the final medical visit in this research. In addition, the PFS was defined as the time period from the time of surgery until the time of disease progression was detected by MRI, CT, or ECT, or the final medical visit. In this study, all experiments were authorized by the hospitals or clinic's ethics board from the Affiliated Changzhou No. 2 People's Hospital, and all patients included in this study were provided with written informed consents.

### 2.2 Analysis of the Gene Expression Profiling Interactive Analysis (GEPIA)

The Gene Expression Profiling Interactive Analysis (GEPIA) (http://gepia.cancer‐pku.cn/) is an online database. In total, data of 9736 tumors and 8587 normal samples from TCGA and GTEx database were included in this website. The correlation analysis module in the database was used to investigate the correlation of given genes. Hence, the correlation analysis (Pearson method) was selected to visualize the co‐expression connection between RNF43 and CD163. On the x‐axis, RNF43 expression was displayed, whereas CD163 expression was shown on the y‐axis.

### 2.3 Immunohistochemistry (IHC)

IHC assay was carried out using UltraSensitive Streptavidin Peroxidase Kit (KIT‐9710, Maixin Biotechnologies) as instructed by the manufacturer. The overnight incubation at 4°C was performed using the primary antibodies listed below: rabbit anti‐RNF43 antibody (1:500, ab217787; Abcam) and rabbit anti‐CD163 (1:200, ab182422, Abcam). A biotin‐labeled secondary antibody and streptomyces antibiotic protein‐eroxidase were then added to the slides for 30 min each. Sections were counterstained with hematoxylin and eosin after staining with Diaminobenzidine (DAB) (DAB‐2031, Maixin Biotechnologies). A score of 0–3 intensity times percent of positive cells (range 0–300) were used to generate an H‐score to evaluate the RNF43 staining. The density of stained stromal immune cells were scored at ×200 magnification through three respective areas of the tumor, and the mean value was accepted. TAMs were counted according to the number of nucleated staining cells in each field. Two independent and authentic pathologists were invited to calculate the score of each patient in double blind way. The ‘survivalROC’ package was adopted to plot Time‐dependent receiver operating characteristic (ROC) curve to determine the optimal cut‐off values of the H‐scores of RNF43 and CD163. Then, H scores greater than the cut‐off values were considered as high expression, in contrast, H score less than cut‐off values represent low expression.

### 2.4 Statistical analysis

R platform (version 4.0.1) was used to perform statistics analyses. The quantitative data were presented as mean and standard deviation (mean ± SD.), while the classified data were expressed as percentages. Quantitative variables were compared using Wilcoxon test or Two‐Tailed Student's *t*‐test, while classified variables were compared using the chi‐square or Fisher's exact test. The predictive performance of RNF43 (H‐score) or CD163 (cells/mm^2^) for 5‐year OS was evaluated using time‐dependent receiver operating characteristic (ROC) curves with the “survival ROC” package, and the optimal cutoff value of RNF43 or CD163 was determined according to the Youden Index. Specifically, the ROC curve was generated by plotting the true positive fraction (sensitivity) on the Y axis versus the false positive fraction (1‐specificity) on the X axis for each RNF43 or CD163 value tested. Then the most applicable cutoff value was defined as the point in the ROC space with the maximum Youden Index, which was calculated as “sensitivity+specificity‐1”. Meanwhile, the area under the ROC curve (AUC) was computed with the “time ROC” package. The log‐rank test was used to compare the survival curves derived using Kaplan–Meier method. Variables with *p*‐value <0.05 by univariate cox regression analysis were embedded in subsequent multivariate analysis (Cox proportional hazards model). *p* < 0.05 was considered as statistic significance. Detailed evaluation of the prognostic prediction models was carried out. The ‘rms’ package was used to generate Harrell's concordance index to evaluate the predictive veracity of RNF43‐classifier and other prognostic signals. Higher c‐index was correlated with better predictive accuracy of survival.

## 3 RESULTS

### 3.1 CD163 ^+^ TAM infiltration in ccRCC patients is adversely correlated with the expression of RNF43

**FIGURE 1:**
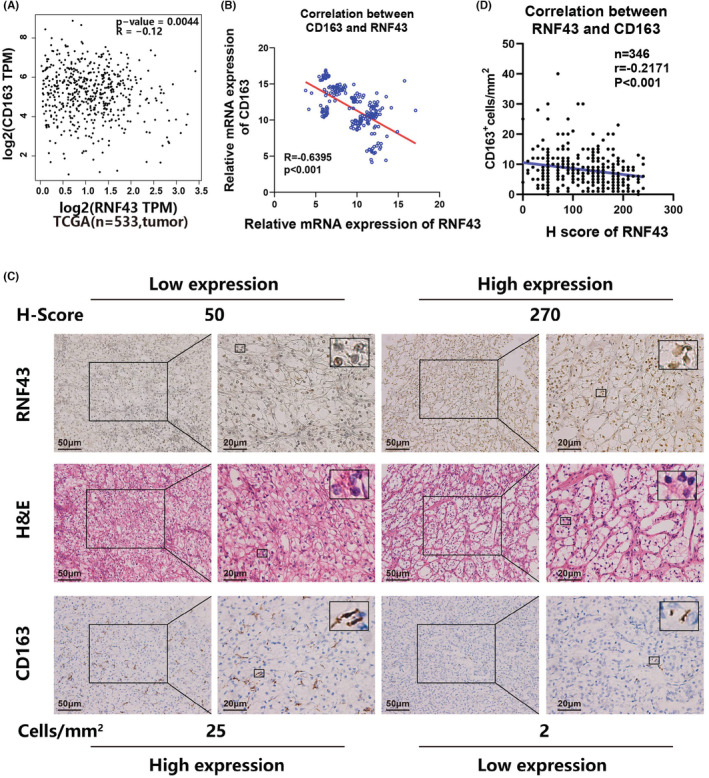
CD163^+^ TAM infiltration in ccRCC patients is adversely correlated with the expression of RNF43. (A) A bioinformatics analysis of Gene Expression Profiling Interactive Analysis (GEPIA)‐derived data was employed to examine the relationship between RNF43 and CD163 expression in ccRCC specimens. (B) The correlation between RNF43 and CD163 based on the mRNA expression via RT‐qPCR (C) Representative images of H&E staining and immunohistochemical (IHC) staining for RNF43 and CD163 in ccRCC are shown (200×, scale bar = 50 μm, 400×, scale bar = 20 μm). (D), A correlation analysis was performed in the relationship between RNF43 and CD163 expression based on the H‐score

We discovered a negative connection between RNF43 and CD163 expressions in ccRCC specimens based on a bioinformatics analysis of Gene Expression Profiling Interactive Analysis (GEPIA)‐derived data. (Figure 1A, *p* = 0.0044). To validate this, RT‐PCR (*n* = 210), and immunohistochemistry (IHC) (*n* = 346) assays were performed to determine RNF43 and CD163 expression in ccRCC specimens, respectively (Figure 1C,D). Results in Figure 1C showed that RNF43 expression was negatively correlated with CD163 expression in tumor samples (*p* < 0.001). Furthermore, as demonstrated in Figure 1D, RNF43 expression was negatively correlated with CD163 expression in tumor samples (*p* < 0.001). These data indicate that there is a negative relevance between the RNF43 expression and CD163^+^ TAMs infiltration level in ccRCC.

### 3.2 Combining expression of RNF43 and CD163 ^+^ TAM infiltration to predict ccRCC patient disease progression and postoperative prognosis

**FIGURE 2:**
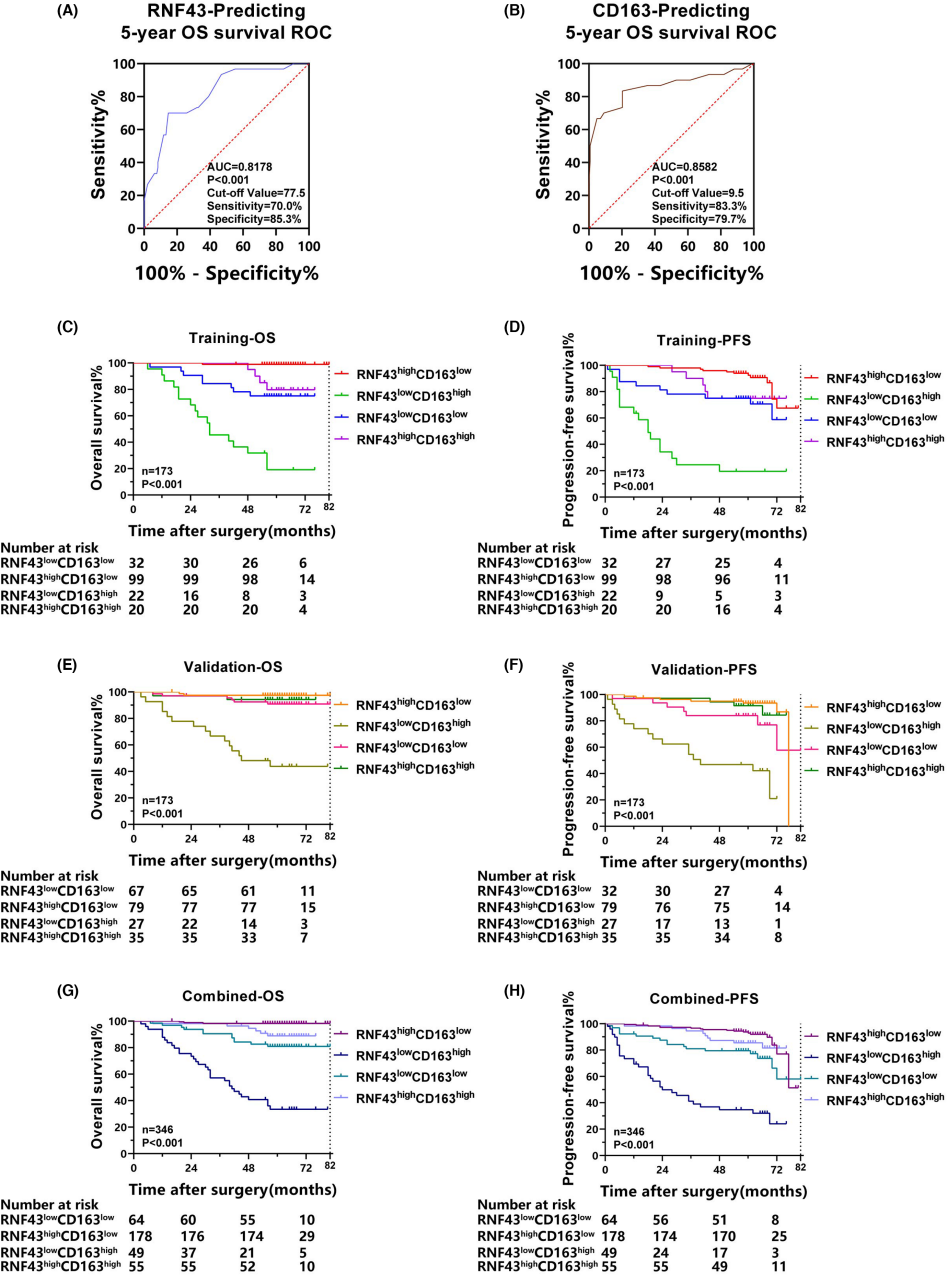
Combining expression of RNF43 and CD163^+^ TAM infiltration to predict ccRCC patient disease progression and postoperative prognosis. (A‐B) A time‐dependent ROC curve analysis was applied to detect the optimum cut‐off values for RNF43 (A) and CD163 (B) in the training cohort. (C‐H) Kaplan–Meier curves showing the OS and PFS of ccRCC patients were generated for the training cohort (C, D), validation cohort (E, F), and combined cohort (G‐H)

To determine whether combining RNF43 expression and CD163^+^ TAM infiltration could predict the postoperative outcome of ccRCC patients. In total, 346 ccRCC patients were enrolled and evenly separated into two datasets. The training cohort and validation cohort contain 173 samples, respectively. Then, IHC assays and ROC curve were performed in the training cohort, which demonstrated that the optimal cutoffs for RNF43 and CD163 were 77.5 and 9.5, respectively (Figure 2A,B **)**. Samples in training cohort were separated into four groups, according to the optimal cutoffs for RNF43 and CD163. Then, the expression of RNF43 or CD163 greater than their cut‐off value was considered as high expression, in contrast, the expression less than the cut‐off value represented low expression. As demonstrated in Table 1, the combined expression pattern of RNF43 and CD163 was closely associated with the TNM stage in the ccRCC patients (*p* = 0.003). Moreover, the group with low RNF43 expression and high CD163 expression presented the worst OS and PFS, but the group with high RNF43 expression and low CD163 expression presented the best OS and PFS (Figure 2C,D **)**. In addition, data in the validation and combined cohorts were used to validate these results. (Figure 2E,H;Tables S2 and S3). Therefore, combining RNF43 expression and CD163^+^ TAM infiltration has the potential to accurately predict PFS and OS in ccRCC patients.

**TABLE 1:** Clinicopathologic characteristics of ccRCC patients by RNF43/CD163 expressions in the training cohort (*n* = 173)

Characteristics	RNF43/CD163 expression				Sum (173)	*p* value
	RNF43^low^	RNF43^high^	RNF43^low^	RNF43^high^		
	CD163^low^	CD163^low^	CD163^high^	CD163^high^		
	(*n* = 32)	(*n* = 99)	(*n* = 22)	(*n* = 20)		
Age						0.709
<60	18	50	9	11	88	
≥60	14	49	13	9	85	
Gender						0.133
Male	27	74	13	17	131	
Female	5	25	9	3	42	
TNM stage						0.003
I‐II	27	94	15	18	154	
III‐IV	5	5	7	2	19	
Overall survival						<0.001
−	24	98	5	16	143	
+	8	1	17	4	30	
Progression free survival						<0.001
−	22	87	5	15	129	
+	10	12	17	5	44	

### 3.3 Integration of RNF43 expression and TAM infiltration with the TNM stage yields superior accuracy in predicting the postoperative outcome of ccRCC

**FIGURE 3:**
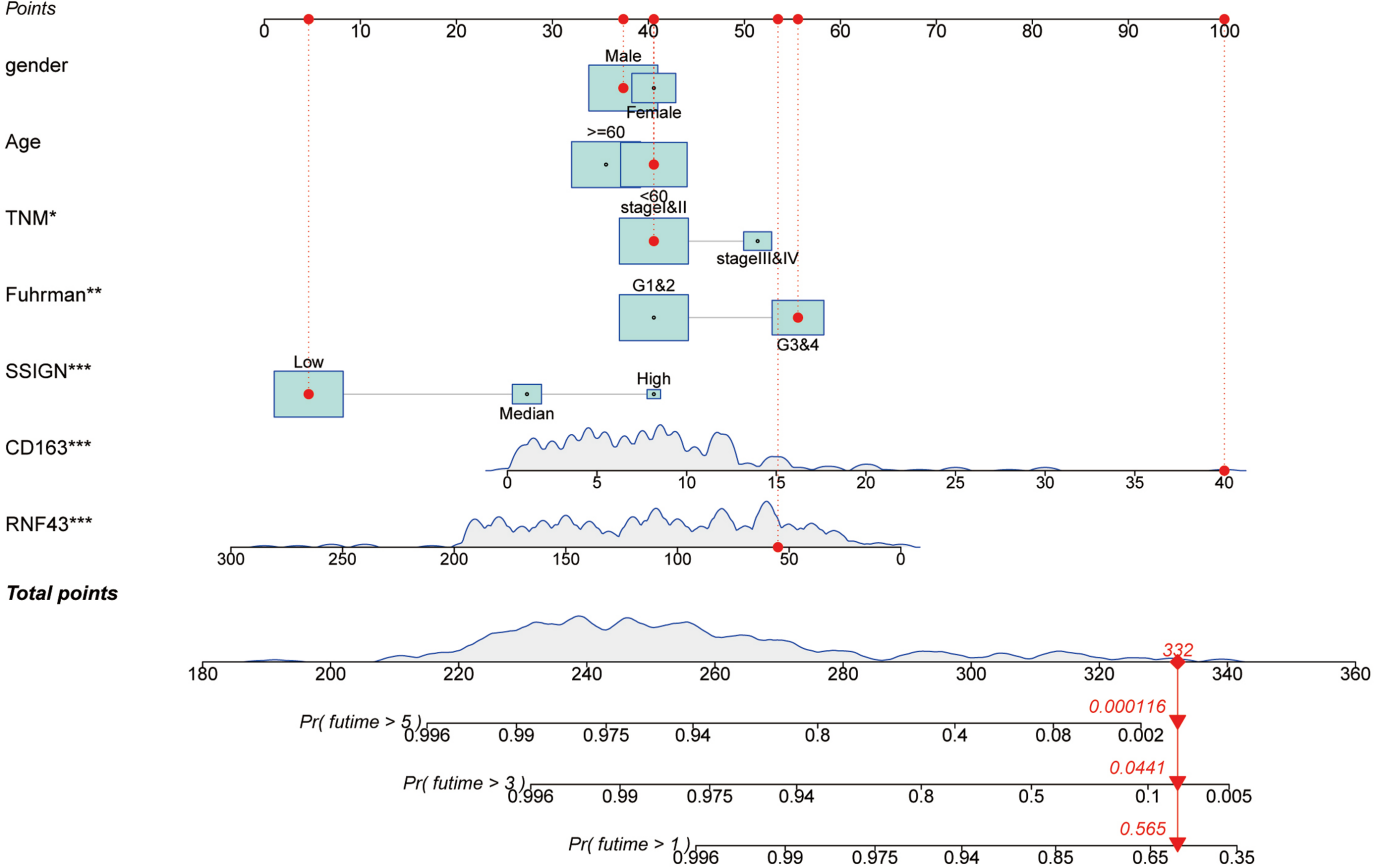
Integration of RNF43 expression and TAM infiltration with the TNM stage yields superior accuracy in predicting the postoperative outcome of ccRCC. A nomogram for the prediction of 1‐, 3‐, 5‐years overall survival in ccRCC patients. Each parameter has a score which could be projected to the Points lines on the top. The total points could be calculated by adding up scores of all parameters

RNF43 expression and CD163^+^ TAM infiltration were further evaluated for their ability to predict the prognosis of patients with ccRCC. Univariate and multivariate Cox regression analyses were performed to probe whether RNF43 expression and CD163 expression could serve as an independent risk indicator. As demonstrated in Table 2 and Tables S4‐S5, after multivariate adjustment, RNF43, CD163, and TNM stage could function as independent risk factors in the training, validation, and combined cohorts. Then, prognostic accuracy of RNF43, CD163, and the currently established indicator TNM stage were compared in accurately predicting ccRCC patient prognosis. As shown in Table 3, time‐dependent c‐index analysis revealed that c‐index values were higher for RNF43 and CD163 combined than for either factor alone, in terms of OS and PFS in ccRCC patients. Moreover, combination the expression of RNF43 and CD163 with the TNM stage presented the highest c‐index value in all comparable groups (Table 3 **)**. These findings imply that integrating RNF43 expression and CD163^+^ TAM infiltration levels with the TNM stage improves the accuracy of predicting the surgical outcome of ccRCC patients. Besides, we further plotted a nomogram for better clinical application of these combined indicators in combined cohort to predict the OS rate at 1‐, 3‐ and 5‐years. Among all included indicators, CD163 expression gained the most significant contribution to survival outcome followed by RNF43 expression. The 1‐, 3‐ and 5‐years survival probabilities of each patient were obtained by adding the score of every prognostic factor (Figure 3).

**TABLE 2:** Univariate and multivariate Cox regression analysis of RNF43/CD163 expression classifier and clinical characteristics with Overall Survival and Progression Free Survival in the training cohort (*n* = 173)

Characteristics	Overall survival				Progression free survival			
	Univariate		Multivariate		Univariate		Multivariate	
	HR (95% CI)	*p* value	HR (95% CI)	*p* value	HR (95% CI)	*p* value	HR (95% CI)	*p* value
Age (≥60 y vs. <60y)	0.79 (0.383–1.626)	0.522			0.857 (0.473–1.552)	0.610		
Gender (Female vs. Male)	1.624 (0.76–3.47)	0.21			1.296 (0.667–2.519)	0.443		
TNM stage (III‐IV vs. I‐II)	8.87 (4.288–18.351)	<0.001	3.407 (1.562–7.431)	0.002	7.774 (4.122–14.661)	<0.001	3.65 (2.221–5.999)	<0.001
RNF43 expression (High vs. Low)	0.107 (0.049–0.234)	<0.001	0.215 (0.093–0.5)	<0.001	0.241 (0.133–0.436)	<0.001	0.391 (0.247–0.619)	<0.001
CD163 expression (High vs. Low)	14.919 (5.699–39.055)	<0.001	9.318 (3.453–25.147)	<0.001	4.822 (2.62–8.873)	<0.001	3.086 (1.874–5.081)	<0.001

**TABLE 3:** C‐index analysis of the prognostic accuracy of RNF43/CD163 classifier and other variables for Overall Survival and Progression Free Survival in training, validation, and combined cohort

Characteristics	Overall survival			Progression free survival		
	Training Cohort (*n* = 173)	Validation Cohort (*n* = 173)	Combined Cohort (*n* = 346)	Training Cohort (*n* = 173)	Validation Cohort (*n* = 173)	Combined Cohort (*n* = 346)
Age	0.535 (0.421–0.648)	0.512 (0.385–0.639)	0.515 (0.431–0.6)	0.509 (0.41–0.608)	0.619 (0.517–0.721)	0.546 (0.474–0.618)
Gender	0.554 (0.437–0.671)	0.557 (0.428–0.686)	0.552 (0.465–0.638)	0.52 (0.42–0.62)	0.584 (0.474–0.693)	0.545 (0.471–0.619)
TNM stage	0.695 (0.575–0.815)	0.7 (0.57–0.83)	0.694 (0.606–0.783)	0.654 (0.551–0.758)	0.703 (0.592–0.813)	0.673 (0.597–0.749)
RNF43 expression	0.776 (0.674–0.878)	0.719 (0.607–0.831)	0.745 (0.67–0.821)	0.672 (0.573–0.771)	0.643 (0.536–0.749)	0.653 (0.581–0.725)
CD163 expression	0.815 (0.729–0.901)	0.779 (0.68–0.879)	0.797 (0.732–0.862)	0.702 (0.607–0.796)	0.708 (0.608–0.809)	0.702 (0.634–0.771)
RNF43 + CD163	0.901 (0.844–0.959)	0.846 (0.753–0.939)	0.873 (0.821–0.925)	0.753 (0.662–0.844)	0.75 (0.649–0.851)	0.746 (0.678–0.814)
RNF43 + CD163 + TNM stage	0.923 (0.866–0.98)	0.87 (0.784–0.955)	0.897 (0.849–0.945)	0.793 (0.705–0.881)	0.824 (0.734–0.913)	0.799 (0.735–0.863)

## 4 DISCUSSION

It is of great significance to search credible indicators for evaluating prognosis and progression of disease in ccRCC patients.^13^ As is known to all, biomarkers of tumors or tumor‐infiltrating immune cells (TIICs) could predict ccRCC patient prognosis. The present study has combined RNF43 expression and CD163^+^ TAM accumulation into a prognostic model with TNM stage, which shows robust accuracy in forecasting the postoperative outcome of ccRCC patients than that achieved with only single indicators.

It is well acknowledged that ring finger protein 43 (RNF43), an E3 ubiquitin ligase, exerts suppressive effects in various cancers, such as colorectal cancer, gastric cancer, and endometrial cancer.^14^, ^15^ RNF43 mutations frequently appear in these cancers, and the downregulation of RNF43 predicts a poor prognosis in patients.^16^ Moreover, our recent study has demonstrated that RNF43 has been served as a tumor suppressor and prognostic biomarker in ccRCC.^11^ Meanwhile, high infiltration of CD163^+^ tumor‐associated macrophages (TAMs) has also been reported to be relevant to poor prognosis in ccRCC, and the interaction between ccRCC and TAMs may contribute to tumor progression.^17^ These findings prompted us to examine whether RNF43 expression was correlated with CD163^+^ TAMs in ccRCC. By analyzing the data from TCGA, performing immunohistochemistry (IHC) and Real‐time PCR assays, a negative correlation between RNF43 and CD163, which was one of the most widely used TAM‐specific biomarkers, was observed in ccRCC samples **(**Figure 1A‐D **)**. Based on these results and considering that biomarkers in ccRCC or TAMs are inadequate to comprehensively reflect tumor heterogeneity, we further combined RNF43 expression and CD163^+^ TAM infiltration to assess patient outcomes after surgery and exhibited superior accuracy than that of RNF43 or CD163 alone.

Meanwhile, the combination of clinical features together with molecular biomarkers could further add value in the prediction of prognosis with more accuracy, and we also incorporated the presence of TNM stage into our newly established model, in order to achieve better prognostic power. Next, Harrell's C‐index a nomogram was used to compare the prediction performance of different indicators or their combinations. As expected, the combination of both RNF43 and CD163 with TNM stage gained the highest accuracy for OS and PFS of ccRCC patients, compared with that of any other indicators alone (Figure 3; Table 3 **)**.

Recently, an increasing number of studies have reported that the interaction network between tumor cells and TAMs promotes the progression of cancers and combining intratumoral markers and TAMs better predicts the disease progression and outcome of patients.^18^ The present study further indicated that the integration of RNF43 expression with TAM infiltration and the TNM stage achieves better accuracy in predicting prognosis than any of single indicators. Besides, a nomogram was generated which predicted the prognosis of patients' outcome more intuitively and conveniently for urologists. It is our hope that the current work will help provide an efficient predictive tool to aid accurate prognostic assessment and guide long‐term follow‐up as well as clinical decision‐making in the era of precision medicine.

But some limitations remain in the present study. As only postoperative ccRCC patients from one single clinical center were enrolled in our study, we will recruit multicentric ccRCC patients who have received tyrosine kinase inhibitor (TKI) or immunotherapy. In addition, why the expression of RNF43 was inversely associated with CD163^+^ TAM infiltration has not been examined. Thus, our future studies will elucidate whether RNF43 mediates the interaction between ccRCC cells and TAMs, which could provide a new target for inhibiting ccRCC progression.

## 5 CONCLUSIONS

The current research reveals that the postoperative prognosis of ccRCC patients may be better predicted by combining intratumoral RNF43 expression and CD163^+^ TAM infiltration with the TNM stage.

## AUTHOR CONTRIBUTIONS

**Dawei Zhu:** Conceptualization (equal); project administration (equal); writing – original draft (equal). **Xiaokai Shi:** Data curation (equal); investigation (equal); writing – original draft (equal). **yi‐jun Tian:** Formal analysis (equal); writing – original draft (equal). **Hao Li:** Data curation (equal). **Bowen Tang:** Formal analysis (equal); investigation (equal). **Ziyi Zhang:** Data curation (equal); software (equal). **Ze Zhang:** Formal analysis (equal); investigation (equal); visualization (equal). **Li Zuo:** Conceptualization (equal); supervision (equal); writing – review and editing (equal).

## FUNDING INFORMATION

The funding for this project came from Changzhou Sci&Tech Program (Scientific and technological support for social development). Grant No. CE20215034.

## CONFLICT OF INTEREST

There are no conflicts of interest to disclose.

## ETHICS APPROVAL

Ethics review boards from the Affiliated Changzhou No. 2 People's Hospital of Nanjing Medical University authorized all experiments, in accordance with the Declaration of Helsinki and the Guidelines for Good Clinical Practice. All ccRCC patients or their legal guardians were provided with written informed consent.

## Supporting information


Tables S1‐S5

## Data Availability

The Pearson correlation analysis was used to visualize the co‐expression relationship between RNF43 and CD163 in Kidney renal clear cell carcinoma (KIRC) samples (*n* = 533) based on the GEPIA (http://gepia.cancer‐pku.cn/) database. All data obtained and analyzed from this website are public and need no further request for usage.
