# Supplementary material for: Combining expression of RNF43 and infiltration level of CD163 + tumor associated macrophage predicts prognosis of clear cell renal cell carcinoma
Source: Cancer Med. 2022 Sep 12;12(4):3962–71. doi: 10.1002/cam4.5229 (PMC9972079; doi:10.1002/cam4.5229)
Supplement: Supplementary file 1 — Tables S1‐S5 [file CAM4-12-3962-s001.docx]

**Table S1 Characteristics of patients with clear cell renal cell carcinoma (ccRCC).**

| Characteristics | Training cohort | Validation cohort | Combined cohort |
| --- | --- | --- | --- |
|  | (n=173) | (n=173) | (n=346) |
| Age |  |  |  |
| ＜60 | 88 | 79 | 167 |
| ≥60 | 85 | 94 | 179 |
| Gender |  |  |  |
| Male | 131 | 115 | 246 |
| Female | 42 | 58 | 100 |
| TNM stage |  |  |  |
| I-II | 154 | 143 | 297 |
| III-IV | 19 | 30 | 49 |
| Overall survival |  |  |  |
| - | 143 | 150 | 293 |
| + | 30 | 23 | 53 |
| Progression free survival |  |  |  |
| - | 129 | 139 | 268 |
| + | 44 | 34 | 78 |

**Table S2 Clinicopathologic characteristics of ccRCC patients by RNF43/CD163 expressions in the validation cohort (n= 173)**

| Characteristic | RNF43/CD163 Expression | | | | Sum(173) | P value |
| --- | --- | --- | --- | --- | --- | --- |
|  | RNF43^low^ | RNF43^high^ | RNF43^low^ | RNF43^high^ |  |  |
|  | CD163^low^ | CD163^low^ | CD163^high^ | CD163^high^ |  |  |
|  | (n=32) | (n=79) | (n=27) | (n=35) |  |  |
| Age |  |  |  |  |  | 0.76 |
| ＜60 | 16 | 36 | 10 | 17 | 79 |  |
| ≥60 | 16 | 43 | 17 | 18 | 94 |  |
| Gender |  |  |  |  |  | 0.445 |
| Male | 21 | 57 | 17 | 20 | 115 |  |
| Female | 11 | 22 | 10 | 15 | 58 |  |
| TNM stage |  |  |  |  |  | 0.001 |
| I-II | 25 | 68 | 16 | 34 | 143 |  |
| III-IV | 7 | 11 | 11 | 1 | 30 |  |
| Overall survival |  |  |  |  |  | <0.001 |
| - | 28 | 77 | 12 | 33 | 150 |  |
| + | 4 | 2 | 15 | 2 | 23 |  |
| Progression free survival |  |  |  |  |  | <0.001 |
| - | 25 | 72 | 11 | 31 | 139 |  |
| + | 7 | 7 | 16 | 4 | 34 |  |

**Table S3 Clinicopathologic characteristics of ccRCC patients by RNF43/CD163 expressions in the combined cohort (n= 346)**

| Characteristic | RNF43/CD163 Expression | | | | Sum(346) | P value |
| --- | --- | --- | --- | --- | --- | --- |
|  | RNF43^low^ | RNF43^high^ | RNF43^low^ | RNF43^high^ |  |  |
|  | CD163^low^ | CD163^low^ | CD163^high^ | CD163^high^ |  |  |
|  | (n=64) | (n=178) | (n=49) | (n=55) |  |  |
| Age |  |  |  |  |  | 0.47 |
| ＜60 | 34 | 86 | 19 | 28 | 167 |  |
| ≥60 | 30 | 92 | 30 | 27 | 179 |  |
| Gender |  |  |  |  |  | 0.292 |
| Male | 48 | 131 | 30 | 37 | 246 |  |
| Female | 16 | 47 | 19 | 18 | 100 |  |
| TNM stage |  |  |  |  |  | <0.001 |
| I-II | 52 | 162 | 31 | 52 | 297 |  |
| III-IV | 12 | 16 | 18 | 3 | 49 |  |
| Overall survival |  |  |  |  |  | <0.001 |
| - | 52 | 175 | 17 | 49 | 293 |  |
| + | 12 | 3 | 32 | 6 | 53 |  |
| Progression free survival |  |  |  |  |  | <0.001 |
| - | 47 | 159 | 16 | 46 | 268 |  |
| + | 17 | 19 | 33 | 9 | 78 |  |

**Table S4 Univariate and multivariate Cox regression analysis of RNF43/CD163 expression classifier and clinical characteristics with Overall Survival and Progression Free Survival in the validation cohort (n=173)**

| Characteristics | Overall survival |  |  |  |  |  | Progression free survival |  |  |  |  |
| --- | --- | --- | --- | --- | --- | --- | --- | --- | --- | --- | --- |
|  | Univariate |  |  | Multivariate |  |  | Univariate |  |  | Multivariate |  |
|  | HR(95%CI) | P value |  | HR(95%CI) | P value |  | HR(95%CI) | P value |  | HR(95%CI) | P value |
| Age（≥60y vs ＜60y) | 0.898(0.524-1.539) | 0.697 |  |  |  |  | 1.321(0.84-2.077) | 0.227 |  |  |  |
| Gender（Female vs Male） | 1.531(0.878-2.668) | 0.132 |  |  |  |  | 1.451(0.913-2.306) | 0.114 |  |  |  |
| TNM stage（3-4 vs 1-2） | 6.601(2.905-14.998) | <0.001 |  | 4.225(1.788-9.983) | 0.001 |  | 6.354(3.216-12.557) | <0.001 |  | 4.859(2.398-9.846) | <0.001 |
| RNF43 expression (High vs Low) | 0.174(0.068-0.442) | <0.001 |  | 0.213(0.083-0.549) | 0.001 |  | 0.321(0.16-0.647) | 0.001 |  | 0.366(0.179-0.75) | 0.006 |
| CD163 expression（High vs Low） | 10.652(3.62-31.336) | <0.001 |  | 6.342(2.045-19.669) | 0.001 |  | 5.148(2.497-10.612) | <0.001 |  | 3.49(1.62-7.519) | 0.001 |

**Table S5 Univariate and multivariate Cox regression analysis of RNF43 expression classifier and clinical characteristics with Overall Survival and Progression Free Survival in the combined cohort (n=346)**

| Characteristics | Overall survival |  |  |  |  |  | Progression free survival |  |  |  |  |
| --- | --- | --- | --- | --- | --- | --- | --- | --- | --- | --- | --- |
|  | Univariate |  |  | Multivariate |  |  | Univariate |  |  | Multivariate |  |
|  | HR(95%CI) | P value |  | HR(95%CI) | P value |  | HR(95%CI) | P value |  | HR(95%CI) | P value |
| Age（≥60y vs ＜60y) | 0.898(0.524-1.539) | 0.697 |  |  |  |  | 1.321(0.84-2.077) | 0.227 |  |  |  |
| Gender（Female vs Male） | 1.531(0.878-2.668) | 0.132 |  |  |  |  | 1.451(0.913-2.306) | 0.114 |  |  |  |
| TNM stage（3-4 vs 1-2） | 7.244(4.215-12.451) | <0.001 |  | 2.898(1.625-5.168) | <0.001 |  | 6.291(3.983-9.934) | <0.001 |  | 3.65(2.221-5.999) | <0.001 |
| RNF43 expression(High vs Low) | 0.142(0.078-0.259) | <0.001 |  | 0.211(0.115-0.387) | <0.001 |  | 0.3(0.191-0.47) | 0.001 |  | 0.391(0.247-0.619) | <0.001 |
| CD163 expression（High vs Low） | 12.586(6.138-25.806) | <0.001 |  | 6.509(3.056-13.863) | <0.001 |  | 4.941(3.107-7.858) | <0.001 |  | 3.086(1.874-5.081) | <0.001 |
